# VZHE-039, a novel antisickling agent that prevents erythrocyte sickling under both hypoxic and anoxic conditions

**DOI:** 10.1038/s41598-020-77171-2

**Published:** 2020-11-20

**Authors:** Osheiza Abdulmalik, Piyusha P. Pagare, Boshi Huang, Guoyan G. Xu, Mohini S. Ghatge, Xiaomeng Xu, Qiukan Chen, Nancy Anabaraonye, Faik N. Musayev, Abdelsattar M. Omar, Jürgen Venitz, Yan Zhang, Martin K. Safo

**Affiliations:** 1 Division of Hematology, The Children’s Hospital of Philadelphia, Philadelphia, PA 19104 USA; 2 Department of Medicinal Chemistry, Virginia Commonwealth University, Richmond, VA 23298 USA; 3 The Institute for Structural Biology, Drug Discovery and Development, School of Pharmacy, Virginia Commonwealth University, Richmond, VA 23298 USA; 4 Department of Pharmaceutics, Virginia Commonwealth University, Richmond, VA 23298 USA; 5 Department of Pharmaceutical Chemistry, Faculty of Pharmacy, King Abdulaziz University, Alsulaymanyah, 21589 Jeddah Saudi Arabia; 6 Department of Pharmaceutical Chemistry, Faculty of Pharmacy, Al-Azhar University, Cairo, 11884 Egypt

**Keywords:** Drug discovery, Structural biology

## Abstract

Sickle cell disease (SCD) results from a hemoglobin (Hb) mutation βGlu6 → βVal6 that changes normal Hb (HbA) into sickle Hb (HbS). Under hypoxia, HbS polymerizes into rigid fibers, causing red blood cells (RBCs) to sickle; leading to numerous adverse pathological effects. The RBC sickling is made worse by the low oxygen (O_2_) affinity of HbS, due to elevated intra-RBC concentrations of the natural Hb effector, 2,3-diphosphoglycerate. This has prompted the development of Hb modifiers, such as aromatic aldehydes, with the intent of increasing Hb affinity for O_2_ with subsequent prevention of RBC sickling. One such molecule, Voxelotor was recently approved by U.S. FDA to treat SCD*.* Here we report results of a novel aromatic aldehyde, VZHE-039, that mimics both the O_2_-dependent and O_2_-independent antisickling properties of fetal hemoglobin. The latter mechanism of action—as elucidated through crystallographic and biological studies—is likely due to disruption of key intermolecular contacts necessary for stable HbS polymer formation. This dual antisickling mechanism, in addition to VZHE-039 metabolic stability, has translated into significantly enhanced and sustained pharmacologic activities. Finally, VZHE-039 showed no significant inhibition of several CYPs, demonstrated efficient RBC partitioning and high membrane permeability, and is not an efflux transporter (P-gp) substrate.

## Introduction

Sickle Cell Disease (SCD) is the most common inherited hematologic disorder affecting between 80,000 and 100,000 people (mostly of black origin) in the U.S. and over 15 million worldwide^1,2^. The number of affected population is projected to increase by 30% by 2050^2^. SCD results from a single-point mutation in hemoglobin (Hb), where βGlu6 of normal Hb (HbA) is changed to βVal6 in sickle Hb (HbS). Under hypoxic conditions or in areas of low partial pressure of oxygen (O_2_), HbS becomes deoxygenated (DeoxyHbS) and polymerizes into long and rigid fibers, causing sickling of red blood cells (RBCs). The low O_2_-affinity of HbS, seemingly due to elevated intra-RBC concentrations of 2,3-diphosphoglycerate (2,3-DPG) and/or sphingosine 1-phosphate (S1P) exacerbates the hypoxia-induced polymerization^3–8^. The rigid sickled RBCs impair blood flow, causing a cascade of interrelated secondary adverse effects. These include, but are not limited to, adhesion of RBCs to tissue endothelium, hemolysis, oxidative stress, decreased vascular nitric oxide (NO) bioavailability, inflammation, painful vaso-occlusion (VOC) crisis, and eventually chronic endothelial and organ damage that ultimately leads to poor quality of life and decreased life expectancy^1,2,9,10^.

While the primary interaction between HbS that initiates the hypoxia-induced polymerization process and the subsequent fiber formation occurs between the pathologic βVal6 residue and a hydrophobic pocket on an adjacent HbS tetramer, the stability of the fiber requires additional secondary interactions between the HbS molecules^11–15^. This is demonstrated by naturally occurring mutations that have been shown to reduce polymerization and sickling by disrupting these contacts^11–18^. For example, αAsn78 → Lys (Hb Stanleyville) and βAsp73 → Val (Hb Mobile) on the surface-located αF-helix of Hb increase the solubility of DeoxyHbS, reducing sickling and lessening the severity of the disease^16–18^.

Until recently, hydroxyurea (HU), which induces fetal Hb (HbF) production had been, for over two decades, the only U.S.-approved drug for the treatment of SCD^19^. The expressed high O_2_-affinity HbF modulates clinical severity by reducing the concentration of HbS to inhibit polymerization, serving as a model for antisickling therapies. A second mechanism of action involves direct destabilization effect of the Hb polymer, as the homo-tetramers of HbF (α_2_γ_2_) and hybrid HbFS tetramers (α_2_γβ^S^) cannot be incorporated into fibers because of disruptive effects on the intermolecular contacts of normal HbS^20^. Three new drugs for SCD were approved in the last three years by the FDA. The first is L-glutamine (Endari), which was approved in 2017^21,22^. l-glutamine works by increasing the amount of reduced form nicotinamide adenine dinucleotide (NADH) in erythrocytes, which is expected to reduce oxidative stress, and potentially result in fewer painful crises and adverse events^23^. In 2019, crizanlizumab (AKA Adakveo)^24^ and Voxelotor^25^ (AKA GBT-440 or Oxbryta) were approved. Crizanlizumab, a monoclonal antibody, reduces the frequency of painful VOCs by targeting P-selectin, which is implicated in the pathologic endothelial adhesion of sickle erythrocytes and leukocytes^24^. Voxelotor is the first aromatic aldehyde-containing antisickling compound approved for SCD that targets HbS polymerization by increasing Hb O_2_-affinity^25–28^. The groundwork for this therapeutic approach began in the 1970s^29^, and was furthered by our group and others with the natural and non-toxic compounds vanillin and 5-HMF, providing the proof-of-principle and roadmap for modern aromatic aldehyde drug candidates^30–40^. The clinical efficacy of Voxelotor for SCD treatment is based on increased Hb levels and reduced hemolysis in patients^25^. Although these surrogate endpoints are not long-term clinical outcomes, the phase III trial provided additional encouraging evidence that aromatic aldehydes may have disease-modifying potential that can mitigate adverse disease effects of RBC sickling.

Hb functions in equilibrium between the “Tense”, deoxygenated T-state and ensemble of “Relaxed”, O_2_-liganded, R-state^38,40–45^. Only in the T-state, DeoxyHbS can polymerize into insoluble fibers, and thus, the kinetics of HbS polymerization and RBC sickling are favored primarily under hypoxic conditions^11^. Aromatic aldehydes, as exemplified by vanillin and 5-HMF, form Schiff-base interactions with the α-subunit N-terminal αVal1 amines in the Hb α-cleft that not only destabilize the T-state, but also stabilize the R-state to increase O_2_-affinity of Hb (pO_2_ at 50% oxygenated Hb, represented as P_50_)^31,34,37,38^. Vanillin and 5-HMF have been studied for their potential to treat SCD^30,34,38,39^, but weak pharmacodynamic (PD) and/or poor pharmacokinetic (PK) properties, in part due to extensive oxidative metabolism of the aldehyde primarily by aldehyde dehydrogenase (ALDH) in the RBCs (target site), and liver^46–48^, have hampered their development into viable SCD therapeutics. Nonetheless, these compounds serve as highly valuable, effective, and non-toxic design scaffolds, as exemplified by the successful development of Voxelotor^25–28^.

Using an iterative, structure-based approach to sequentially improve the inherent design of aromatic aldehyde-containing vanillin analogs^30–38,40,41^, we have identified a novel aromatic aldehyde, VZHE-039, that not only shows improved PK/PD properties but demonstrates unique antisickling properties. While the antisickling effects of aromatic aldehydes, such as Voxelotor, are mainly dependent on modulating Hb O_2_ affinity, Hb tetramers modified by VZHE-039 like HbF resist sickling not only due to increasing O_2-_affinity, but also by weakening polymer intermolecular contacts (O_2_-independent antisickling effect) that are critical to the stability of insoluble fibers. We expect that this novel aromatic aldehyde and/or analogs will be developed as novel therapeutic to treat SCD patients.

## Results

### Design and synthesis of VZHE-039

**Figure 1:**
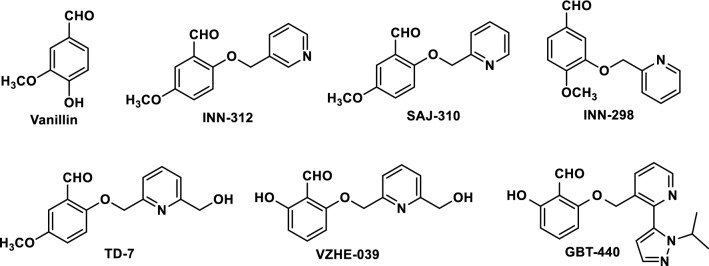
Structures of aromatic aldehydes.

We have previously demonstrated through structural studies, that two molecules of the naturally occurring antisickling aromatic aldehydes, vanillin or 5-HMF preferentially bind at the α-cleft of liganded Hb in the R2-state conformation, form Schiff-base interactions with αVal1 amines to increase the protein affinity for oxygen, reduce hypoxia-induced HbS polymerization and RBC sickling^34^. Based on the crystallographic binding of vanillin, we systematically modified the structure of vanillin to implement additional interactions with the α-cleft of Hb to increase potency. In the first iterative step, we incorporated methoxy-pyridine (methoxyPy) onto the aromatic ring of vanillin, resulting in INN and SAJ derivatives, *e.g*., SAJ-310, INN-312, INN-298 (Fig. 1)^31,33,35,36^. These molecules engaged in additional, enhanced interactions with the protein, and potently modified Hb to increase O_2_-affinity and inhibit RBC sickling^31,33,35,36^. With the methoxyPy substituted at the *ortho*-position of the aldehyde, e.g., INN-312 or SAJ-310, we established that the new derivatives made weak hydrophobic interactions with the surface-located F helix of α globin (αF-helix)^31,35^ that led to moderate increases in the solubility of completely deoxygenated HbS through an *O*_*2*_*-independent* antisickling mechanism^31^. Such a mechanism is distinct from (and complementary to) the primary mechanism of increasing Hb affinity for O_2_ (i.e. *O*_*2*_*-dependent* antisickling mechanism). Further supporting this line of thought is the knowledge that the αF-helix is involved in polymer stabilization through αAsn78 mediated hydrogen-bonding interactions with other polymer strands, as demonstrated by the antisickling properties of Hb Stanleyville^16–18^ We therefore hypothesized that increased perturbation of the orientation of the αF-helix would improve the *O*_*2*_*-independent* antisickling activities of the molecules. Based on this novel concept, we engaged in new rounds of *in-silico* modelling and structure-based design to introduce potentially stronger contacts with the protein and αF-helix by incorporating a methylhydroxy moiety on the pyridine ring and/or increase the metabolic stability of the aldehyde moiety, leading to the discovery of several compounds, e.g. TD-7 and VZHE-039 (Fig. 1), with the latter being the most promising with unique antisickling activity. The study with TD-7 has been published^33^ and will be used for comparative purpose in this manuscript.

**Scheme 1:**
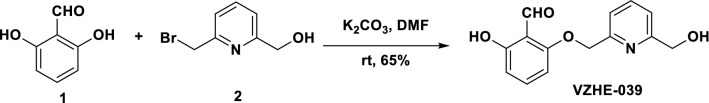
Synthetic route for the synthesis of VZHE-039

VZHE-039 was prepared as outlined in Scheme 1, and detailed synthesis is described in the experimental section. In brief, molar equivalents of commercially available 2,6-dihydroxybenzaldehyde (1) and 6-(bromomethyl)-2-pyridinemethanol (2) were reacted under mildly basic conditions at room temperature, and the product (VZHE-039) precipitated as an off-white solid with addition of water. Subsequent recrystallization resulted in a fine white powder (58% yield, 97% purity). VZHE-039 was used for crystallographic and several studies as outlined below.

### VZHE-039 binds to Hb as designed with enhanced interactions with Hb and the αF-helix

**Figure 2:**
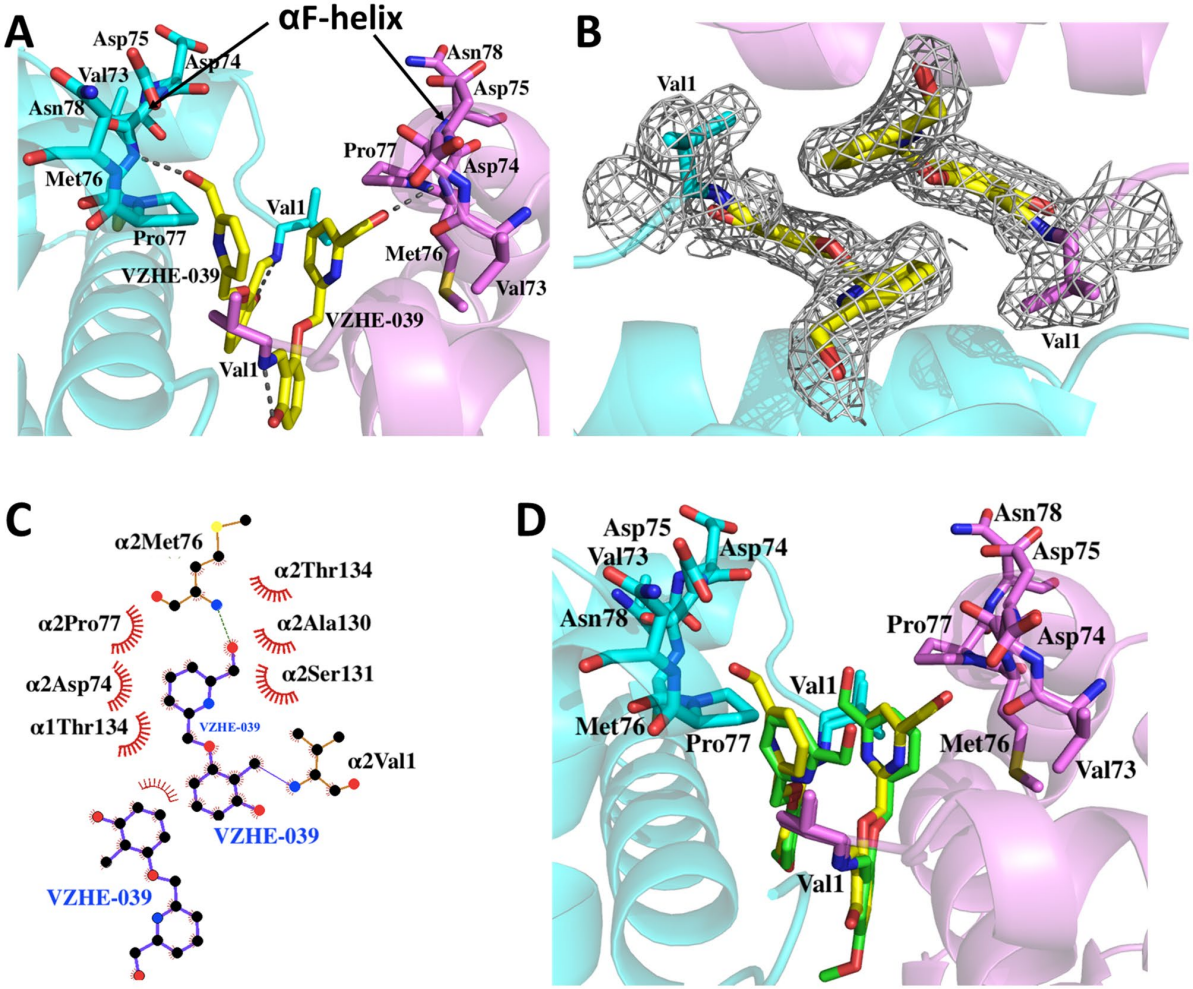
Structure of Hb in the R2 conformation in complex with two molecules of VZHE-039 bound at the α-cleft. For clarity, not all binding site residues are shown, but described in the text. Hb subunits are shown as ribbons (α1 subunit in pink, α2 in cyan). (**A**) A pair of bound VZHE-039 (yellow sticks) at the α-cleft of Hb showing the close hydrogen-bond interaction with the nitrogen atom of Met76 of the αF-helix. (**B**) Final 2F_o_-F_c_ electron density map of VZHE-039 (yellow stick) contoured at 1.0σ. (**C**) Two-dimensional contacts between one VZHE-039 molecule, the protein, and the second VZHE-039 molecule as described in the text. (**D**) Superposition of TD-7 (green) and VZHE-039 (yellow) molecules at the α-cleft of Hb. (**A**–**D**) were generated using the Pymol graphic software (The Pymol Molecular Graphics System, version 1.7.4, Schrödinger, LLC; https://pymol.org/2/support.html). (**C**) was generated with LIGPLOT: a program to generate schematic diagrams of protein–ligand interactions, version 2.2 (https://www.ebi.ac.uk/thornton-srv/software/LigPlus/)^49^.

One of our major objectives was to develop compounds that would bind at the α-cleft with stronger interactions with the protein and αF-helix, which we hypothesize, would lead to novel O_2_-independent antisickling activity. To ascertain whether VZHE-039 binds as predicted, we determined its crystal structure in complex with liganded Hb in the R2 conformation. Detailed crystallographic data are summarized in Table 1, with the atomic coordinates and structure factors deposited in the RCSB Protein Data Bank as entry 6XD9. As expected and similar to previous aromatic aldehydes (e.g., INN-312, TD-7, and SAJ-310), two molecules of VZHE-039 bound in a symmetry-related fashion at the α-cleft of the Hb tetramer with the aldehyde moieties forming Schiff-base interactions with the two αVal1 N-terminal amines (Fig. 2). The benzaldehyde ring made both intra- and inter-hydrophobic interactions with α2Ser131 and α1Thr134. The Schiff-base interaction directed the methoxyPy group toward the αF-helix residue to make moderate intrasubunit hydrophobic interactions (3.5–4.5 Å) with the helix (Fig. 2A). The pyridine rings also showed extensive 3.7–4.0 Å π-π interactions with each other. It is interesting to note that, unlike TD-7 or VZHE-039 that binds two pairs of the respective molecules at the α-cleft of Hb, the bulkier Voxelotor only binds one molecule of the compound due to steric reason^28^.

**Table 1:** Crystallographic data and refinement statistics for VZHE-039 bound Hb complex.

Data collection statistics	
Space group	P2_1_2_1_2_1_
Unit-cell *a, b, c* (Å)	62.78, 83.63, 105.00
Resolution (Å)	29.34- 2.1 (2.18- 2.1)
Unique reflections	31,379
Redundancy	4.62 (4.47)
Completeness (%)	96.3 (95.40)
Average I/σ(I)	12.4 (3.9)
R_merge_ (%)^a^	6.8 (34.2)
**Refinement statistics**	
Resolution (Å)	29.34–2.1
No. of reflections	31,350
R_work_ (%)	19.72
R_free_ (%)^b^	25.82
R.m.s.d. bonds (Å)	0.003
R.m.s.d. angles (°)	0.696
Dihedral angles	
Most favored (%)	96.11
Allowed (%)	3.71
Average B (Å^2^)/atoms	
All atoms	31.00
Macromolecule	30.70
VZHE-039	28.00
Water	35.30

^a^*R*_merge_ = Σ_*hkl*_Σ_i_|*I*_i_(*hkl*) − < *I*(*hkl*) >|/Σ_*hkl*_Σ_i_*I*_i_(*hkl*)*.*

^b^R_free_ was calculated from 5% randomly selected reflection for cross-validation. All other measured reflections were used during refinement.

Consistent with our design prediction, VZHE-039 made novel and strong hydrogen-bond interaction with the αF-helix using the methylhydroxy moiety. This bond interestingly was missing in TD-7 structure, despite the presence of a methylhydroxy moiety in TD-7. In the VZHE-039 structure, the methylhydroxy forms a strong hydrogen bond interaction (2.9 Å) with the backbone nitrogen atom of αMet76 of the αF-helix (Fig. 2A). TD-7 does not make such a hydrogen-bond interaction with the αF-helix due to apparent ~ 180° rotation of the pyridine ring from that of VZHE-039 (and away from the αF-helix) (Fig. 2D). VZHE-039, like other aromatic aldehydes, e.g. TD-7 further makes weak hydrophobic interactions with the αF-helix. In another structural difference, the benzaldehyde hydroxyl moiety of VZHE-039 (which is absent in TD-7) is engaged in a strong hydrogen-bond interaction with αVal1 nitrogen (Fig. 2A,C). Most likely, this interaction may have led to the differences in the positioning of the pyridine methylhydroxy moiety in the two structures, which in VZHE-039 allows close interaction between the methylhydroxy and the αF-helix. The increased interactions between VZHE-039 and the protein, particularly the αF-helix are expected to directly destabilize the polymer, as well as translate into increased biological potency, which were studied and described below.

### VZHE-039 demonstrated sustained pharmacologic effect in vitro

**Figure 3:**
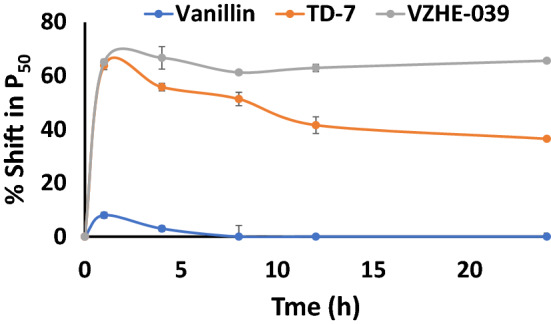
Time-dependent P_50_-shift of HbA in normal blood incubated with 2 mM test compound.

The antisickling activity of aromatic aldehydes is dependent on Schiff-base interaction between the aldehyde moiety and the Hb αVal1 amines (Schiff-base adduct) and the increase in the protein affinity for oxygen^30–40^. Unfortunately, the aldehyde function is highly susceptible to oxidative metabolism into inactive carboxylic acids, mainly by NAD-dependent aldehyde dehydrogenase (ALDH) in the RBC and liver^46–48^, resulting in lower concentrations at the Hb molecule, reducing binding and shortening the duration of their antisickling effects. To overcome this pharmacologic limitation, Tucaresol and Voxelotor, two potent antisickling agents utilized a *ortho*-hydroxyl moiety (relative to the aldehyde group) on the benzaldehyde ring to form intramolecular hydrogen-bond interaction with the aldehyde group to form hemiacetal-like moiety, which led to significant reduction in oxidative metabolism of the aldehyde^28,50^. Consistently, TD-7 or vanillin or 5-HMF, without the *ortho*-hydroxyl protective group, showed significant metabolism of the aldehyde^32,33^. VZHE-039, however, incorporates this *ortho*-hydroxyl moiety on the benzene ring, which is expected to increase its oxidative metabolic stability. This was tested by incubating 2 mM of VZHE-039 (with vanillin and TD-7 as controls) with fresh normal adult whole blood (Hct of 30%) at 37 °C as a surrogate measure of oxidative metabolism. At defined time points (1, 4, 8, 12 and 24 h), aliquot samples were drawn, and subsequently analyzed for their P_50_-shifts relative to the initial P_50_ value, using three-point tonometry. In parallel, an untreated control sample was also assessed to control for any time-dependent, but drug-independent changes in P_50_ values. Samples incubated with VZHE-039 showed a sustained P_50_-shift throughout the entire 24-h experimental period (Fig. 3). TD-7, on the other hand, showed a maximum effect at 1 h, followed by a gradual decrease in effect to ~ 45% reduction in activity at 24 h. Vanillin did not have any effects beyond 4 h. These findings confirm the importance of the *ortho*-hydroxyl group in aromatic aldehydes in preventing or reducing enzymatic metabolism of aldehyde in RBCs. Although, similar *in-vitro* time-dependent P_50_-shift studies were not reported for Tucaresol and Voxelotor, these molecules showed prolonged duration of action with half-life values  > 12 h in experimental animals and humans^28,50–52^.

### VZHE-039 potently increased Hb adduct formation and Hb O_2_-affinity, as well as improved antisickling activity in vitro

**Figure 4:**
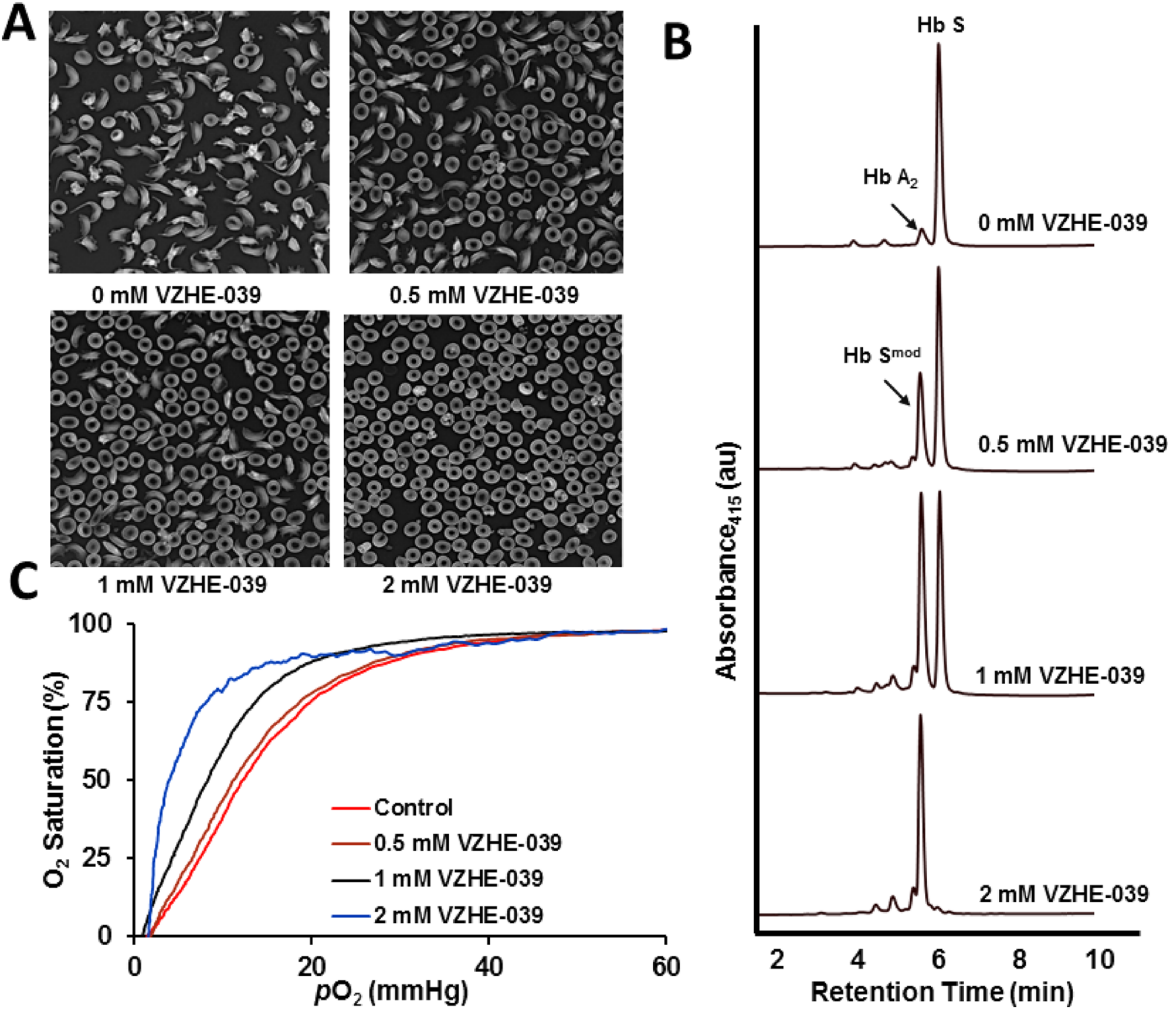
Dose-dependent effect of VZHE-039 on SS cells sickling, Hb adduct formation and Hb O_2_-affinity in vitro (Hct of 20%). (**A**) Morphology of SS cells before and after incubation with VZHE-039 under 2.5% O_2_. (**B**) Representative HPLC chromatograms (Hb modification analyses) of lysates from the antisickling study. (**C**) Representative OEC curves of lysates from the antisickling study.

For our previously studied compounds (e.g., INN-312, TD-7, SAJ-310, INN-298) that incorporate methoxyPy substitution to vanillin analogs, we observed a significant increase in functional and biological activity as a result of increased protein interactions^31,33,35,36^. We therefore expected similar effects for VZHE-039, which we tested by following VZHE-039 activity (measured by Hb adduct formation, P_50_-shift and RBC sickling inhibition) using SS blood. Briefly, we incubated 0.5, 1, and 2 mM concentrations of VZHE-039 with whole blood suspensions from subjects with homozygous SCD (Hct of 20%) under hypoxic conditions (2.5% O_2_/97.5% N_2_) at 37 °C for 2 h for expected peak adduct. Aliquot blood samples were drawn into a fixative (2% glutaraldehyde solution), and sickling was assessed by microscopy^31,33,35,36^. Aliquot samples were also subjected to cation-exchange HPLC analyses to assess the degree of Hb modification to the high-affinity adduct form (Hb adduct formation), as well as standard O_2_ equilibrium curves (OEC) to assess P_50_-shifts using hemox analyzer^31,33,35,36^. The results summarized in Table 2, and Fig. 4 demonstrate the concentration-dependent inhibition of SS cell sickling and the corresponding modification of HbS (HbS^mod^); both endpoints correlated linearly with the observed P_50_-shifts (left shift in OEC). When compared with the previously observed functional/biological effects with TD-7^33^, VZHE-039 showed better PD effects, especially at lower concentrations. For, example, at 0.5 mM, 1 mM and 2 mM, TD-7 inhibited RBC sickling by 16%, 29%, and 85%, respectively (compared to 35%, 63%, and 93% for VZHE-039); showed Hb modification of 26%, 45%, and 74% (compared to 38%, 72%, and 98% for VZHE-039); and demonstrated P_50_-shifts of 10%, 28%, and 48% (compared to 22%, 47%, and 76% for VZHE-039). In comparison, at 0.5 mM and 1.0 mM concentrations, Voxelotor decreased RBC sickling by 56% and 98%, modified Hb by 55% and 100%, and increased Hb 41% and 80%, respectively, suggesting that Voxelotor shows better *direct* potency than VZHE-039. The improved potency of Voxelotor has been attributed to reduced binding stoichiometry, i.e. while Voxelotor binds Hb at a 1:1 ratio, VZHE-039 binds Hb at a 2:1 ratio. It is of interest that unlike TD-7 and Voxelotor, VZHE-039 shows a weaker-than-expected correlation between the antisickling effect and P_50_ shift. We speculate, that the relatively low P_50_ shifts (22%, 47%, and 76% at 0.5 mM, 1.0 mM, and 2.0 mM, respectively) do not fully account for the substantial antisickling effects (35%, 63%, and 93%, respectively). This observation, as will be discussed later is likely due to a meaningful contribution from the novel O_2_-independent antisickling activity as result of the strong contact with the αF-helix.

**Table 2:** Hemoglobin modification, change in O_2_-equilibrium, and antisickling studies of VZHE-039 using human sickle blood.

Test	% Functional/biological effect		
	0.5 mM	1 mM	2 mM
Sickling Inhibition^a^	34.5 ± 2.4	63.7 ± 6.9	92.8 ± 5.5
Hb Modification^b^	38.0 ± 7.2	72.6 ± 6.4	97.4 ± 6.7
P_50_ Shift^c^	22.3 ± 8.7	46.6 ± 5.4	75.6 ± 2.5

All studies were conducted with SS cells suspensions (20% hematocrit) incubated with 0.5, 1 and 2 mM of VZHE-039. The results are the mean values ± SD for 6 individual replicate experiments. The final concentration of DMSO was < 2% in all samples, including in control samples.

^a^Antisickling studies with SS cells were conducted under hypoxia (2.5% Oxygen).

^b^Hb S adduct values obtained from HPLC elution patterns of individual hemolysates after incubation of compounds with SS cells.

^c^P_50_ is the oxygen pressure at which the hemolysates are 50% saturated with oxygen. ΔP_50_ (%) was determined as:$\Delta {\text{P}}_{50} (\% ) = \frac{{{\text{P}}_{50} \;{\text{of}}\;{\text{lysates}}\;{\text{from}}\;{\text{untreated}}\;{\text{cells}} - {\text{P}}_{50} \;{\text{of}}\;{\text{lysates}}\;{\text{from}}\;{\text{treated}}\;{\text{cells}}}}{{{\text{P}}_{50} \;{\text{of}}\;{\text{lysates}}\;{\text{from}}\;{\text{untreated}}\;{\text{cells}}}} \times 100$.

### VZHE-039 exhibited a novel O_2_-independent antisickling mechanism that is distinctly different from its primary mechanism of increasing Hb O_2_-affinity

**Figure 5:**
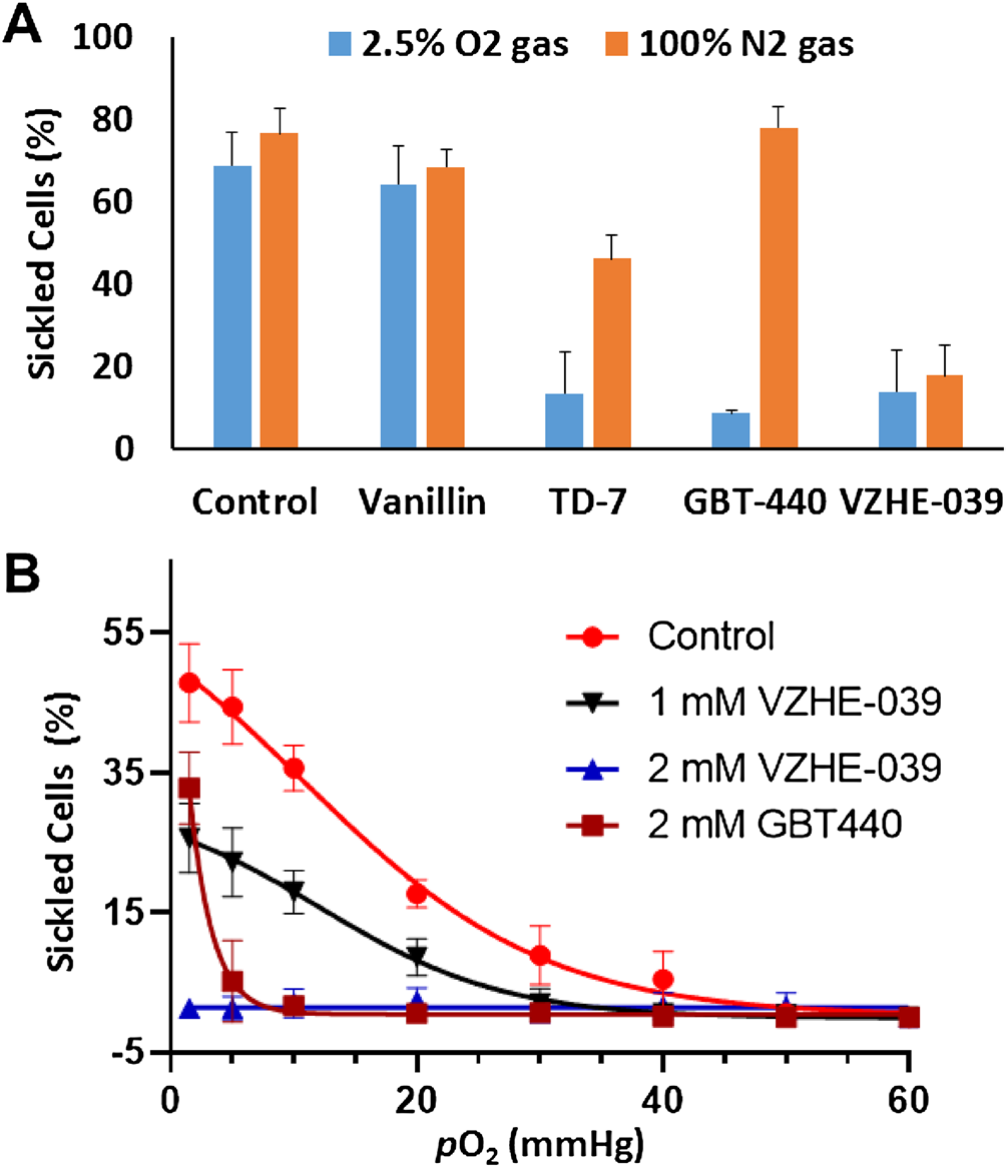
Antisickling effects of VZHE-039 on sickling of SS cells under various conditions. (**A**) Comparison of inhibition of SS cell sickling under 2.5% O_2_ gas and 100% N_2_ gas, at 2 mM concentrations, for untreated (control), Vanillin, TD-7, GBT-440 (Voxelotor), and VZHE-039. We observe identical antisickling effects for VZHE-039 under either hypoxia or anoxia, conclusively demonstrating an O_2_-independent effect, which is not present or much less prominent for the comparator compounds. (**B**) *p*O_2_-dependent sickling of SS RBCs under controlled conditions in a Hemox Analyzer (Control, VZHE-039 and Voxelotor. Note the delay in initiation of sickling, as well as reduction in the total number of sickled cells at 1 mM concentration, while a complete inhibition of sickling at the lowest recorded *p*O_2_ is seen at 2 mM for VZHE-039.

Our previous studies with VZHE-039 precursors, including INN-312 and SAJ-310 suggested weak interactions between these compounds and the αF-helix that translated into weak O_2_-independent antisickling activity^31,35^. Therefore, another objective in the targeted design of VZHE-039 was to complement its O_2_-dependent antisickling activity—common to this class of aromatic aldehydes—with an improved O_2_-independent antisickling activity through proximity-enhanced auxiliary interactions with the αF-helix. Findings from the VZHE-039 crystallographic studies indicate that we achieved the structural objective of enhanced interactions between the methylhydroxy substituent and the αF-helix. We further asked whether these enhanced interactions would translate to an improvement in the O_2_-independent antisickling activity—as hypothesized. We therefore explored the potential O_2_-independent activity of VZHE-039, by testing its antisickling properties under complete deoxygenated/anoxic conditions (i.e., 100% N_2_). We used vanillin, TD-7 and Voxelotor as reference controls, the latter known to have the most potent in vitro P_50_-shift and antisickling activity in the presence of O_2_^25–28^. For internal control purposes, aliquots from samples used for testing for antisickling studies at 2.5% O_2_ (see above) were also used for testing the antisickling effect of these compounds under 100% N_2_ gas, therefore, helping elucidate the antisickling effects due to O_2_-dependent activity of the compounds. Since this experimental design utilized aliquots of the same samples under different gas conditions, it ensured that the assay was free of any potential errors associated with variability in hematocrit and accuracy in drug concentrations. As shown in Fig. 5A, the most striking observation was that VZHE-039 inhibited sickling under complete anoxia (100% N_2_ gas) while Voxelotor lost its inherent antisickling effects remarkably under anoxic conditions despite its complete Hb modification to the high-O_2_-affinity form. Although TD-7 showed some antisickling effect under anoxia, it was significantly less potent than VZHE-039, confirming the added benefit of our targeted structural modification. As expected, vanillin showed no effect under anoxic conditions.

In a confirmatory study, a pO_2_ dependent degree of sickling of VZHE-039 at 1 and 2 mM doses, with Voxelotor as a control was performed using a Hemox analyzer. As expected from the above result, VZHE-039 (2 mM) showed complete sickling inhibition at all pO2, and a significant delay in initiation of sickling at 1 mM (Fig. 5B). In contrast, Voxelotor (2 mM) despite its ability to completely prevent sickling at high pO2 (Fig. 5B), lost this capability at low pO2. Oksenberg et al.reported similar observations with Voxelotor^28^, where the compound lost its potent antisickling activity at low pO_2_ values. These experimental findings are consistent with the crystallographic findings: VZHE-039 demonstrated the most proximate and strongest interactions with the αF-helix (2.9 Å), while the other compounds, TD-7 and Voxelotor, showed only weak hydrophobic interactions (3.5–4.0 Å) and no interaction at all as observed with vanillin (> 4.2 Å)^28,33^.

### VZHE-039 partitions efficiently into the RBC compartment, and has acceptable ADME/safety characteristics

**Figure 6:**
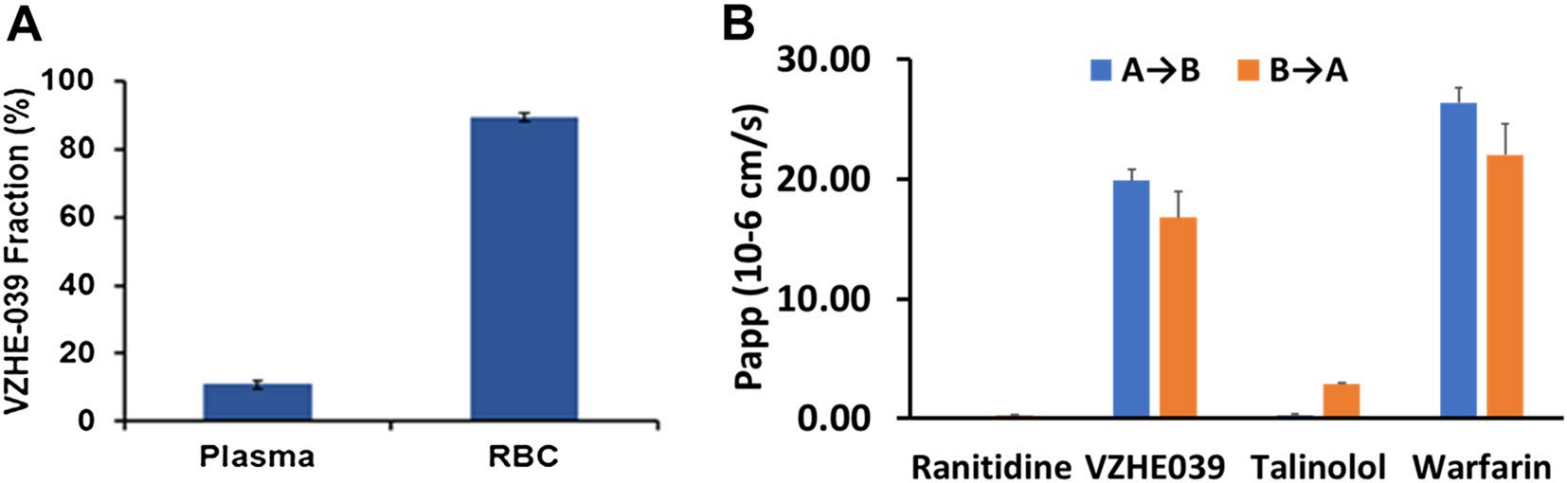
RBC partitioning and cell permeability by VZHE-039. (**A**) Partitioning of VZHE-039 into RBC compartment (n = 4) (**B**) Bi-directional permeability through caco-2 cell monolayers.

Toxicity concerns remain an important issue in the use of aromatic aldehydes in treating a chronic disease, such as SCD, especially as it involves modification of a large amount of in vivo present Hb. Experimental and clinical data from Voxelotor and 5-HMF, however, have demonstrated that when administered at therapeutically efficacious doses in vivo*,* both compounds partition into the RBC compartment and bind to Hb with high specificity^28,30^, likely mitigating possible off-target binding concerns. We evaluated key metrics such as in vitro RBC partitioning and ADME/safety profiles, in order to glean important safety insights. When we incubated VZHE-039 at blood concentrations, expected to be biologically relevant (100–300 µM) with normal whole human blood at 37 °C, after which the compounds concentrations were quantitated by HPLC–MS in plasma, whole blood and RBCs, our results showed that approximately 85% of VZHE-039 partitioned into the RBC compartment across the range of concentrations measured (Fig. 6A), which compares with 90% partitioning by Voxelotor^28^. These findings demonstrate the ability of both compounds to reach their biological target, i.e., Hb, despite any plasma protein binding that may prevent RBC partitioning. We also assessed VZHE-039 at 10 μM for its bi-directional cell permeability using Caco-2 cell monolayers^53,54^. The results demonstrated high in vitro GI permeability of VZHE-039 without involvement of efflux transporters (efflux ratio [R_e_] of 0.85) (Fig. 6B), which may be predictive of acceptable oral bioavailability, absent limitations from GI solubility and/or first-pass metabolism^53^.

Next, we tested VZHE-039 at concentrations of 0.1 to 100 µM, considered to be biologically relevant unbound plasma concentrations, for possible CYP inhibition using pooled human liver microsomes and isozyme-specific probe substrates (CYP1A2, CYP2C8, CYP2C9, CYP2C19, CYP2D6, CYP3A4, CYP2B6)^55^. VZHE-039 showed no significant CYP inhibition (IC_50_ > 100 µM for most CYPs) with the exception of CYP1A2 that showed inhibition at 4.7 µM (Table 3). The result compares favorably with that of Voxelotor^26^.

**Table 3:** In vitro CYP Inhibition with VZHE-039.

Enzyme	Substrate	IC_50_ (μM)	
		Control Inhibitor	VZHE-039
CYP1A2	Tacrine	α-Naphthoflavone (0.005)	4.7
CYP2C8	Amodiaquine	Quercetin (1.2–1.75)	> 100
CYP2C9	Tolbutamide	Sulfaphenazole (0.129–0.205)	> 100
CYP2C19	S-Mephentoin	Ticlopidine (1.4)	> 100
CYP2D6	Dextromethorphan	Quinidine (0.035–0.163)	> 100
CYP3A4	Midazolam	Ketoconazole (0.018–0.0234)	> 100
CYP3A4	Testosterone	Ketoconazole (0.015–0.019)	95.0
CYP2B6	Bupropion	Ticlopidine (0.250)	41.4

## Discussion

Several important investigations for novel therapies for SCD, which either target the primary pathophysiology of hypoxia-induced Hb polymerization and/or various secondary pathologic pathways are currently undergoing development or approved for treating SCD. It is recognized that, due to the inherently complex downstream pathophysiology and the phenotypic heterogeneity of SCD, a single therapeutic mode may not be universally beneficial^56,57^. As an exception and a model, HU has multiple modes of therapeutic action, including the ability to prevent hypoxia-induced RBC sickling, inhibit platelet aggregation, decrease inflammation, and, most importantly, ameliorate painful VOC^20,58,59^. Through targeted structure-based drug design, we have discovered a novel antisickling agent, VZHE-039, that forms Schiff-base adducts with Hb and exhibits both O_2_-dependent and O_2_-independent antisickling mechanisms of action. The O_2_-dependent antisickling effect, which is a significant improvement on our earlier lead compound TD-7^33^, is due to additional interactions with the Hb protein, and the protection of the aldehyde group from untoward rapid metabolism inside the RBC. More importantly, the enhanced O_2_-independent antisickling effect is due to close interactions with the Hb polymer-stabilizing αF-helix. This dual antisickling effect of VZHE-039 is expected to improve its pharmacologic effect in vivo – by targeting both the primary pathophysiology of the disease, Hb polymerization, by two different mechanism, one of which is operative even in the absence of oxygen, which may be quite relevant at low pO2 values in capillaries of SCD patients. This expectation is based on individuals of Sudanese and Congolese ancestry who inherit the rare double mutant Hb variant, referred to as HbS Stanleyville, possessing the pathogenic HbS (βGlu6 → βVal6) mutation and a second (αAsn78 → αLys78) mutation on the αF-Helix^16–18^. Similar to individuals with hereditary persistence of fetal hemoglobin (HPFH), inheritance of this variant significantly improves the clinical phenotype of SCD, with significantly fewer sickling episodes^16,17^. Critical to the formation and stability of the fiber is αAsn78-mediated intermolecular interaction, and substitution to a positively charged Lys as in HbS Stanleyville strongly inhibits polymerization^16,17^. Comparable to Hb Stanleyville, VZHE-039′s unique interactions with the αF-helix likely perturbs its orientation and moves important stabilizing contacts, including αAsn78 out of register, resulting in direct Hb polymer destabilization. Consequently, unlike traditional aromatic aldehydes whose antisickling activity is based on O_2_-dependent effects only, VZHE-039 possesses both the O_2_-dependent and O_2_-independent antisickling properties of HbF tetramers, which will be crucial to treat various clinical phenotypes. While aromatic aldehyde-bound HbS tetramers may still transition to the DeoxyHb T-state in areas of severe regional hypoxia and be incorporated into polymer fibers, VZHE-039-bound DeoxyHb T-state HbS would continuously destabilize fiber formation. Finally, the novel dual mechanism of action of VZHE-039 may deliver enhanced antisickling potency beyond the practical limitations of increasing O_2_ affinity, which is inherently limited by the need to avoid impeding O_2_ unloading to tissues^60^.

Another unique property of VZHE-039 is that unlike our previous aromatic aldehydes, e.g., vanillin, 5-HMF and TD-7, but similar to Voxelotor or Tucaresol, it demonstrates resistance to oxidative metabolism inside the RBC due to protection by the phenylhydroxy moiety, leading to sustained and improved pharmacologic activities in vitro (and potentially in vivo). VZHE-039 also showed no significant inhibition of several CYPs, demonstrated efficient RBC partitioning, high GI permeability—all of which may be predictive of acceptable oral bioavailability, absent limitations from GI solubility and/or first-pass metabolism as well as a low liability of metabolic drug-drug interactions. A combination of these attributes strongly suggests that VZHE-039 has the potential to confer potent antisickling effects with improved clinical outcomes.

## Conclusions

Through a methodical and targeted drug discovery approach, we have identified an important high O_2_-affinity antisickling agent, VZHE-039, that, in addition to Hb modification, disfavoring the polymer forming Deoxy Hb, directly inhibits Hb polymerization and RBC sickling under anoxia. This O_2_-independent antisickling mechanism is likely to permit the in vivo prevention of sickling without drastically changing O_2_ tissue delivery, making VZHE-039 a potential and possibly superior candidate for treating SCD, compared to other related aromatic aldehydes. This is critical for a disease that is characterized by severe hypoxia. In summary, VZHE-039 represents one of the most promising chemotypes, with improved PD characteristics that have translated into significantly enhanced and sustained pharmacologic activities in vitro*,* in addition to acceptable in vitro ADME and safety properties.

## Methods

### Study approvals

At Virginia Commonwealth University (VCU), normal whole blood was collected from adult donors (> 18 years) after informed consent, in accordance with regulations of the IRB for Protection of Human Subjects (IRB# HM1) by the VCU Human Research Protection Program/Institutional Review Board. Leftover blood samples from patients with homozygous SS were obtained and utilized, based on an approved IRB protocol (IRB# 11-008151) by the Committees for the Protection of Human Subjects of the Institutional review board at the Children’s Hospital of Philadelphia. All experimental protocols and methods were performed in accordance with institutional (VCU and CHOP) regulations.

### Materials and general procedures

All reagents used in the syntheses and functional assays were purchased from Sigma-Aldrich (St. Louis, MO) and ThermoFisher Scientific (Waltham, MA) and mostly utilized without additional purification. Voxelotor (GBT-440) was purchased from MedChemExpress (Monmouth Junction, NJ).

### Synthesis of 2-((6-(Hydroxymethyl)pyridin-2-yl)methoxy)-6-hydroxybenzaldehyde, VZHE-039

A mixture of 2,6-dihydroxybenzaldehyde (276 mg, 2.0 mmol), 6-(bromomethyl)-2-pyridinemethanol (404 mg, 2.0 mmol), and K_2_CO_3_ (276 mg, 2.0 mmol) in anhydrous DMF (4 mL) was allowed to stir from 0 °C to room temperature for 15 h. Water (50 mL) was added, and the off-white solid was precipitated. The solid was filtered, dried, and dissolved in methanol (30 mL). The solution was refluxed with active charcoal for 20 min. After filtration, the solution was concentrated, and the resulted solid was crystallized from methanol to give fine powder (300 mg, 58%). Mp: 109.5–110.0 °C. ^1^H NMR (400 MHz, DMSO-*d*_6_) *δ*: 11.75 (s, 1H, Ph-OH), 10.41 (s, 1H, CHO), 7.86 (t, *J* = 7.72 Hz, 1H, Py-H), 7.54–7.44 (m, 3H, Ph-H, Py-H), 6.68 (d, *J* = 8.32 Hz, 1H, Ph-H), 6.55 (d, *J* = 8.40 Hz, 1H, Ph-H), 5.47 (t, *J* = 5.36 Hz, 1H, OH), 5.28 (s, 2H, CH_2_), 4.58 (d, *J* = 4.84 Hz, 2H, CH_2_). ^13^C NMR (100 MHz, DMSO-*d*_6_) *δ*: 193.86, 162.40, 161.63, 160.90, 154.88, 138.72, 137.58, 119.50, 119.35, 110.71, 109.57, 103.39, 70.89, 64.09. IR (Diamond, cm^-1^): 3177, 2860, 1640, 1622, 1600, 1577, 1446, 1399, 1370, 1347, 1317, 1295, 1274, 1238, 1197, 1160, 1107, 1066, 1006, 998, 904, 839, 769, 726, 675. ESI–MS: *m/z* 282.0751 [M+Na]^+^, C_14_H_13_NO_4_ (259.0845). Purity as determined by HPLC: 97%.

### X-ray crystallography

Freshly made solution of VZHE-039 in DMSO was added to DeoxyHb (30 mg/mL protein) at an Hb tetramer-compound ratio of 1:10, followed by saturation with carbon monoxide and allowed to incubate for 2 h to form COHb-compound complex. Sodium cyanoborohydride (NaBH_3_CN) was then added to this mixture to reduce the Schiff-base adduct formed between the protein and compound to the corresponding irreversible alkylamine covalent bond. The resulting solution was crystalized using 10–20% PEG 6000, 100 mM HEPES buffer, pH 7.4 using the batch method as previously published^33^. Single cherry red needle crystals were formed in 1–3 days and were used to collect x-ray diffraction data at 100 K using Rigaku MicroMax™ 007HF X-ray Generator, Eiger R 4 M Detector and Oxford Cobra Cryo-system (The Woodlands, TX). The crystals were first cryoprotected with 80 μL mother liquor mixed with 62 μL of 50% PEG6000. The diffraction data was processed using d*trek software (Rigaku) and the CCP4 suite of programs. The crystal structure of the complex was solved by a molecular replacement method with the Phenix program^61,62^, using the native R2-state crystal structure (PDB ID 1BBB) as a search model. The structure was refined using both Phenix and CNS while model building and correction was carried out using COOT^61–63^. The atomic coordinates and structure factors of VZHE-039 in complex with liganded Hb are deposited in the RCSB Protein Data Bank as entry 6XD9.

### In vitro time-dependent Hb oxygen equilibrium studies using normal whole blood

Normal whole blood samples (hematocrit 30%) in the absence (control) or presence of 2 mM concentration VZHE-039 (solubilized in DMSO) were incubated at 37 °C for 24 h with shaking (at 140 rpm). At 1, 4, 8, 12 and 24 h time intervals, aliquots of this mixture were removed and then subjected to OEC analysis using tonometry as previously described^34^. Vanillin and TD-7 were used as positive controls, while DMSO was tested as negative control. Briefly, the compound-treated blood samples were incubated in IL 237 tonometers (Instrumentation Laboratories, Inc. Lexington, MA) for approximately 10 min at 37 °C, and allowed to equilibrate at oxygen tensions 6, 20, and 40 mmHg. The samples were then aspirated into an ABL 700 Automated Blood Gas Analyzer (Radiometer) to determine the pH, partial pressure of CO_2_ (pCO_2_), partial pressure of oxygen (pO_2_), and Hb oxygen saturation values (SO_2_). The measured values of pO_2_ (mmHg) and SO_2_ at each pO_2_ value were then subjected to a non-linear regression analysis using the program Scientist (Micromath, Salt Lake City, UT) to estimate P_50_ as previously reported^34^.

### Hemoglobin modification, oxygen equilibrium and antisickling studies using human sickle blood

The effect of VZHE-039 on RBC sickling, Hb modification, and Hb oxygen equilibrium was studied utilizing samples from consented individuals with homozygous SCD following previous procedure^33^. Briefly, blood suspended in Hemox buffer and supplemented with glucose and bovine serum albumin, to a final hematocrit of 20% were incubated under air in the absence or presence of 0.5 mM, 1 mM and 2 mM VZHE-039 at 37ºC for 1 h. This was followed by incubating the suspensions under hypoxic conditions (2.5% O_2_/97.5% N_2_) at 37ºC for 2 h. Aliquot samples were obtained and fixed with 2% glutaraldehyde solution without exposure to air, and then subjected to microscopic morphological analysis of bright field images (at × 40 magnification) of single layer cells on an Olympus BX40 microscope fitted with an Infinity 2 camera (Olympus), and the coupled Image Capture software. Leftover samples were washed in phosphate-buffer saline, and hemolyzed in hypotonic lysis buffer for subsequent Hb-modification and oxygen equilibrium analyses.

For the oxygen equilibrium studies, 100 μL aliquot samples from the above clarified lysate were added to 4 mL of 0.1 M potassium phosphate buffer, pH 7.0, in cuvettes and subjected to hemoximetry analysis using a Hemox Analyzer (TCS Scientific Corp.) to determine the P_50_ values.

Finally, for the Hb adduct formation studies, the above clarified lysates were subjected to cation-exchange HPLC (Hitachi D-7000 Series, Hitachi Instruments, Inc., San Jose, CA), using a weak cation-exchange column (Poly CAT A: 30 mm × 4.6 mm, Poly LC, Inc., Columbia, MD). A commercial standard consisting of approximately equal amounts of composite HbF, HbA, HbS and HbC (Helena Laboratories, Beaumont, TX), was utilized as the reference for isotypes. The areas of new peaks, representing HbS adducts, were obtained, calculated as percentage fractions of total Hb area, and reported as levels of modified Hb.

### Antisickling activities of VZHE-039 under anoxia

We conducted two studies to test the antisickling property of VZHE-039 under anoxia to establish the secondary mechanism of action. In the first study, we conducted antisickling studies as described above and also previously reported (2.5% O_2_)^33^, using 2 mM concentrations of VZHE-039, and the controls vanillin, TD-7 and Voxelotor. After 1 h, aliquot samples were fixed with 2% glutaraldehyde without exposure to air. Then the incubation chamber was opened and exposed to air for 15 min to ensure complete re-oxygenation and reversal of the sickled cells to normal round cells. Reversal was confirmed by microscopy. The incubation chamber was then closed, and the assay was repeated under 100% nitrogen gas for 30 min, at which point aliquots were obtained and fixed. Aliquot samples were then subjected to microscopic morphological analysis of bright field images as previously described^33^. Resulting sickled cells (percentages) were compared across samples, and between aliquots of the same samples that had been obtained either under 2.5% oxygen or 100% nitrogen.

The second study established pO_2_ dependent degree of sickling of VZHE-039, with Voxelotor as a control. Concurrently, blood samples, hematocrit 20%, were incubated without any drug (control) or with 1, or 2 mM VZHE-039 at 37 °C for 1 h. A second control sample was incubated with 2 mM Voxelotor. At conclusion, 100 uL aliquots of each suspension were mixed with 2.5 mL Hemox buffer (pH 7.4, supplemented with antifoam), and transferred into the sample chamber of the Hemox Analyzer. Compressed air was then flushed through the sample to ensure complete oxygenation (at 150 mmHg). Nitrogen gas was then introduced into the sample chamber, and 50 uL aliquots were obtained and fixed at defined pO_2_ values. At conclusion, all fixed samples were subjected to microscopic analysis for their degree of sickling, and results were plotted using Prism Graph Pad software. The experiments were conducted in three replicates on different days, and mean and standard deviation values are reported.

### In vitro CYP-450 inhibition studies

VZHE-039 was studied for its inhibitory potential of seven major drug metabolizing human cytochrome P450 (CYP) enzymes (CYP1A2, CYP2C8, CYP2C9, CYP2C19, CYP2D6, CYP3A4, CYP2B6) using pooled human liver microsomes as published previously^26,55^. The probe substrates include tacrine (CYP1A2), amodiaquine (CYP2C8), tolbutamide (CYP2C9), mephenytoin (CYP2C19), dextromethorphan (CYP2D6), midazolam (CYP3A4), testosterone (CYP3A4), and bupropion (CYP2B6). The following selective CYP inhibitors, naphthoflavone (CYP1A2), quercetine (CYP2C8), sulfaphenazole (CYP2C9), ticlopidine (CYP2C19), quinidine (CYP2D6), ketoconazole (CYP3A4), and ticlopidine (CYP2B6) were used as positive controls. Assay conditions were optimized for each human cytochrome P450 substrate. The optimized reaction mixtures (200 μL) contained a final concentration of 0.2–0.5 mg/mL pooled human liver microsomes, 2 mM NADPH in 100 mM potassium phosphate, pH 7.4 buffer with 5 mM MgCl_2_, and VZHE-039 concentration of 0.1 to 100 µM. The assays were performed in duplicate in 96-well plates at 37 °C for 10–60 min. The reaction was terminated with addition of methanol, followed by incubation at 4 °C for 10 min and centrifuged at 4 °C for 10 min. Effect of VZHE-039 on formation of the respective probe substrate metabolites (velnacrine, N‐desethylamodiaquine, 4-hydroxytolbutamide, 4′-hydroxymephenytoin, dextrophan, 1′-hydroxymidazolam, and 6β-hydroxtestosterone and hydroxybupropion) were determined using LC–MS/MS. Metabolite of each CYP was used to calculate IC_50_ value, which is the VZHE-039 concentration that resulted in 50% inhibition.

### Caco-2 permeability experiments

Human epithelial colorectal adenocarcinoma (Caco-2) cells (HTB-37) were cultured in T75 flasks using complete Dulbecco’s Modified Eagles Medium (DMEM) containing 10% fetal bovine serum (FBS), 1% glutamine, 1% penicillin and 1% streptomycin, at 37 °C in a 5% CO_2_ atmosphere. Cells were passaged at 80–90% confluency using 0.05% trypsin–EDTA and the medium was changed every other day. Following this, the cells were trypsinized, suspended in medium and applied to a Millipore 96-well plate where they were cultured as monolayers at a density of 25,000 cells/well. The cells were incubated in a 37 °C/5% CO_2_ incubator to allow cell attachment and proliferation. Media was changed every 2–3 days for 21 days when cells reached 100% confluency. For Apical → Basolateral (A → B) permeability, 10 μM VZHE-039 was added to the apical (A) side and the amount of permeation determined on the basolateral (B) side; for Basolateral → Apical (B → A) permeability, 10 μM VZHE-039 was added to the B-side and the amount of permeation was determined on the A side. The A-side buffer contained 100 μM lucifer yellow dye, in Transport Buffer (1.98 g/L glucose in 10 mM HEPES, 1 × Hank’s Balanced Salt Solution) pH 7.4, and the B-side buffer used was the Transport Buffer at pH 7.4. Caco-2 cells were incubated with 10 μM VZHE0-39 in these buffers for 2 h. Ranitidine (low permeability), Warfarin (high permeability) and Talinolol (P-gp efflux control) were used as controls. At the end of the assay, donor and receiver side solution samples were collected, quenched by 100% methanol containing an internal standard and centrifuged at 5000 rpm for 10 min at 4 °C. Following centrifugation, the supernatant for donor and receiver side samples was analyzed by LC–MS/MS to determine peak area ratios.

Data was expressed as (P_app_):

$$P_{app} = \frac{{{{dQ} \mathord{\left/ {\vphantom {{dQ} {dt}}} \right. \kern-\nulldelimiterspace} {dt}}}}{{C_{0} A}}$$

where dQ/dt is the rate of permeation, C_0_ is the initial concentration (10 μM) and, A is the area of the monolayer.

The efflux ratio (R_e_) was calculated as:

$${\text{R}}_{{\text{e}}} = \frac{{P_{app} \left( {B \to A} \right)}}{{P_{app} \left( {A \to B} \right)}}$$

### Potential of VZHE-039 to partition into RBCs

We incubated test compounds at expected blood concentrations (100–300 uM) with whole blood (mouse or human), after which we analyzed compound concentrations separately in plasma, whole blood and RBCs. Previously, we have established and validated reversed-phase HPLC and LC/MS methods for accurately measuring the compounds in blood. Validated reversed-phase HPLC was conducted on a Hitachi system, using a Waters C18 column, and a gradient of 0.1% Formic Acid in water (mobile phase A), and 0.1% Formic Acid in Acetonitrile (mobile phase B) at a flow rate of 1 mL/min in 12 min. UHPLC and LC/MS analyses were conducted at the Bioanalytical Core Facility at The Children’s Hospital of Philadelphia. A Waters Oasis PRiME HLB µElution Plate was utilized for SPE preparation of samples for HPLC/UHPLC analysis, and consistent recovery of VHZE-039 (a dynamic range of 10–50,000 ng/mL) was achieved. The UHPLC method used the AB Sciex fixed needle injector, on a Phenomenex Kinetex F5 2.6 um 100 A, 4.6 × 50 mm column. 5 uL of each sample was injected at analyzed using a gradient of: (A) 5 mM ammonium acetate in deionized water (pH adjusted to 4.8 by formic acid); and (B) 5 mM ammonium acetate in (90:10) acetonitrile: water, at a flow rate of 0.5 mL/minute. Mass spectrometry was conducted on an AB Sciex 6500 QTRAP mass spectrometer. VZHE-039 assay has a dynamic range from 10 to 50,000 ng/mL using 10 µL of sample.

## Data Availability

The atomic coordinates and structure factors of VZHE-039 in complex with liganded Hb are deposited in the RCSB Protein Data Bank as entry 6XD9.
